# Increased Antigen Presentation but Impaired T Cells Priming after Upregulation of Interferon-Beta Induced by Lipopolysaccharides Is Mediated by Upregulation of B7H1 and GITRL

**DOI:** 10.1371/journal.pone.0105636

**Published:** 2014-08-21

**Authors:** Fang Wang, Yan Yan Wang, Juan Li, Xiang You, Xin Hui Qiu, Yi Nan Wang, Feng Guang Gao

**Affiliations:** 1 Department of Immunology, Medical College, Xiamen University, Xiamen, China; 2 Department of Basic Medicine Science, NanYang Medical College, Nanyang, China; Istituto Superiore di Sanità, Italy

## Abstract

Dendritic cells are able to present Ag-derived peptides on MHC class I and II molecules and induce T cells priming. Lipopolysaccharides (LPS), an activator of Toll-like 4 receptor (TLR4) signaling, has been demonstrated to facilitate Ag-presentation, up-regulate surface molecules expression but impair T cells priming. In this study, we investigated the effect of LPS on nicotine-enhanced DCs-dependent T cells priming and the mechanisms of LPS orchestrating the immunosuppressive program. We could demonstrate that the treatment with LPS resulted in increased surface molecules expression, enhanced Ag-presentation, up-regulated release of TGF-beta, TNF-alpha, IL-6, and IFN-beta. Concomititantly, the upregulation of IFN-beta in DCs induces the up-regulation of coinhibitory molecules B7H1 and GITRL, which cause an impaired activation of naïve Ag-specific T cells and the induction of T cell tolerance by enhancing B7H1-PD-1 interactions and promoting GITRL-GITL facilitated Treg generation, respectively. These data provide a mechanistic basis for the immunomodulatory action of IFN-beta which might open new possibilities in the development of therapeutic approaches aimed at the control of excessive immune response and persistent infection.

## Introduction

Dendritic cells (DCs) internalize extracellular antigens and present Ag-derived peptides in the context of MHC molecules to T cells, indicating that DCs are professional APCs which play an important role in the induction of adaptive immune responses [1]. Both classical Ag presentation and cross-presentation enable DCs to activate Ag-specific cytotoxic CD8^+^ T cells which are definitely important for anti-tumor or anti-virus immune responses [2], [3]. Nicotine, a major component of cigarette smoke which promotes established tumor metastasis and increases overall mortality in cancer patients [4], [5], is widely accepted as a risk factor for carcinogenesis and atherosclerosis [4]. Although nicotine could promote lung cancer development, reduce the efficacy of chemotherapeutic agents [6] and activate hypoxia-inducible factor-1 α expression [7], our and others previous studies demonstrated that nicotine treatment activates bone marrow-derived DCs [8] and nicotine-enhanced DCs’ cross-presentation have potential antitumor effects [9]–[11]. Further studies revealed that although both nicotine and lipopolysaccharides (LPS) treatments up-regulate surface co-stimulator molecules expression [12], the treatment with LPS enable DCs to present Ags in the context of MHC I molecules but that they are refractory to CD8^+^ T cells priming [13]–[15]. Hence, to date, the exact effect and mechanism of LPS on nicotine-enhanced DCs’ presentation have not been fully characterized and remains controversial.

Type I interferon (IFN-I), which triggers protective defenses against viral infection, are proteins released by host cells in response to the presence of pathogens such as viruses, bacteria, parasites. As an upstream of hundreds of inflammatory genes, IFN-I is responsible for persistent lymphocytic choriomeningtis virus (LCMV) infection [16], whereas interfering with chronic IFN-I signaling during persistent LCMV infection redirect the immune environment to enable control of infection [17], indicating that IFN-I signaling might contribute to DCs’ immunosuppressive program[14]. Meanwhile, the immune tolerance induced by LPS triggering Toll-like receptor (TLR) signaling in macrophages was also documented [18]. However, to date, little is known about how and to what extent LPS treatment contributes to the mechanisms orchestrating the immunosuppressive program of DCs, which is an important issue for potential therapeutic molecules discovery to overcome persistent infection.

Regulatory T cells (Treg), which express CD4, CD25 and Foxp3 molecules, are a subpopulation of T cells in modulating immune system, maintaining tolerance to self-antigens, and abrogating autoimmune disease [19]–[20]. Kole A *et al* demonstrated that IFN-I regulate regulatory T cell accumulation and anti-inflammatory cytokine production during T cell-mediated colitis [21]. Meanwhile, the treatment with IFN-beta facilitating Treg proliferation through up-regulating GITRL expression was also documented [22]. Our previous studies also showed that TGF-beta present in the microenvironment of lung cancer upregulates GITRL expression and is associated with Treg generation [20]. But, up to now, the exact roles of Treg in LPS-induced DCs paralysis are still unknown.

In the present study, we investigated the effect of LPS on nicotine-enhanced DCs’ presentation which subsequently activates T cells priming and the mechanisms of LPS orchestrating the immunosuppressive program. We demonstrated both *in vitro* and *in vivo* that up-regulation of IFN-beta lead to increased surface co-stimulator molecules expression and presentation of Ags but concomitantly impaired activation of T cells due to increased signaling via the coinhibitory molecules B7H1 and GITRL.

## Materials and Methods

### Reagents

Reagents were purchased from the following companies: nicotine, LPS and ovalbumin (OVA) were obtained from Sigma-Aldrich (St. Louis, MO, USA). Recombinant murine GM-CSF, IL-4 and TGF-beta were obtained from R&D (Minneapolis, MN, USA). RPMI 1640 medium and fetal bovine serum (FBS) were purchased from Hyclone (Logan, UT, USA). H-2K^b^ CTL peptide of OVA (SIINFEKL) was synthesized by Sangong, (Shanghai, China). BrdU cell proliferation kit and IFN-gamma Elispot kit were obtained from Roche (Basel, Switzerland) and U-CyTech Biosciences (Utecht, Netherlands) respectively; Recombinant murine IL-6, TNF-alpha, Brefeldin A solution and Fluorescein-conjugated antibodies to CD4, CD25, Foxp3, CD80, CD86, CD40, OX40L, 4-1BBL, MHC class I, MHC class II, CD11c, TGF-beta, TNF-alpha, and IL-6 were obtained from eBioscience (San Diego, CA, USA). Recombinant Mouse IFN-beta, IFN-beta neutralizing antibody (MIB-5E9.1), B7H1 neutralizing antibody (10F.9G2), fluorescein-conjugated antibodies to IFN-beta, SIINFEKL-H2K^b^, B7H1, GITRL, GITR were purchased from Biolegend (San Diego, CA, USA). The NF-kappaB inhibitor PDTC and Bay 11-7082 were purchased from Cayman Chemical (Ann Arbor, MI, USA). TRI-zol was purchased from Invitrogen life technologies (Carlsbad, CA, USA). SYBR Premix Ex Taq and PrimeScript Reverse Transcriptase were purchased from Tarkara (Dalian, Liaoning, China).

### Animals

Pathogen-free C57BL/6 mice (female, 6–8 weeks old) were bought from the Shanghai Laboratory Animal Center of Chinese Academy of Sciences (China) and kept at the Animal Center of Xiamen University. Animals were housed with an inverse 12 hours day-night cycle with lights on at 8∶30 pm in a temperature and humidity controlled room. All cages contained wood shavings, bedding and a cardboard tube for environmental enrichment. This study was carried out in strict accordance with the recommendations in the Guide for the Care and Use of Laboratory Animals of the ARRIVE guidelines. The protocol was approved by the Committee on the Ethics of Animal Experiments of the Xiamen University. All surgery was performed under sodium pentobarbital anesthesia, and all efforts were made to minimize suffering.

### Generation of murine bone marrow-derived DCs

Bone marrow-derived DCs were generated as previously described [11]. Briefly, bone marrow mononuclear cells were prepared from bone marrow suspensions by depletion of red cells and then cultured at a density of 1×10^6^ cells/ml in RPMI 1640 medium supplemented with 10% FBS at the presence of 10 ng/ml recombinant murine GM-CSF and 1 ng/ml recombinant murine IL-4. Non-adherent cells were gently washed out with PBS on day 4 of culture; the remaining loosely adherent clusters were used as DCs. Cells were synchronized by serum starvation (in RPMI 1640 with 0.5% FBS) for 6 h before further treated with LPS or recombinant murine cytokines.

### DCs treatment for flow cytometry

To determine the effect of nicotine, LPS or IFN-beta on surface molecules expression, cytokine secretion and Treg priming, DCs were exposed to nicotine (10^−7^ M), LPS (10 ng/ml) or IFN-beta (10 ng/ml) for 12 h and then determined via flow cytometry or real-time PCR, respectively. The pretreatment with 5 µg/ml αIFN-beta neutralizing antibody (MIB-5E9.1) 2 h prior to 12 h LPS (10 ng/ml) treatment was done to investigate the role of IFN-beta in LPS-induced DCs paralysis. To elucidate the mechanism of LPS-induced IFN-beta expression, DCs were firstly blocked with the kinase inhibitor PDTC (40 µM) or Bay 11-7082 (20 µM) for 2 h. Then, the DCs were further conferred 12 h LPS (10 ng/ml) exposure.

### DCs treatment for mixed lymphocyte reaction

To investigate the effect of LPS on DCs-dependent T cell proliferation, DCs (cultured for 4 d) were pretreated with LPS (10 ng/ml), IFN-beta (10 ng/ml), TGF-beta (1 ng/ml), TNF-alpha (20 pg/ml) or IL-6 (20 pg/ml) for 12 h prior to 4 h OVA peptide SIINFEKL pulse at the concentration of 2 µg/ml. To determine the role of IFN-beta or B7H1 in LPS-induced DCs paralysis, DCs were pretreated with αIFN-beta (5 µg/ml) (MIB-5E9.1) or αB7H1 (5 µg/ml) (10F.9G2) neutralizing antibodies prior to OVA peptide SIINFEKL peptide (2 µg/ml) pulse. Then, the DCs were cocultured with splenocytes of same background C57BL/6 mice.

### Surface antigens flow cytometric measurements

Surface molecules expression in DCs was determined via flow cytometry according to the methods described previously [11]. Briefly, to block non-specific Fc-mediated interactions, DCs were firstly pre-incubated with 0.5 µg of anti-mouse CD16/CD32 antibodies for 10 min at room temperature prior to staining. Aliquot cell suspension to the well and combined the recommended quantity of each primary antibody in an appropriate volume of flow cytometric staining buffer. Staining was performed on ice for 30 min and cells were washed with ice-cold phosphate-buffered saline (PBS), containing 0.1% NaN3 and 0.5% BSA. Flow cytometry was performed using a FACSCalibur flow cytometer, and the data were analyzed using CellQuest software.

### Intracellular cytokines flow cytometric measurements

To determine the effect of LPS or IFN-beta on cytokines expression, DCs (cultured 4 d) were treated with LPS (10 ng/ml) or IFN-beta (10 ng/ml) for 12 h and brefeldin A solution was added to inhibit protein transport 6 h prior to flow cytometric measurements. Cytokines expression in DCs was determined via flow cytometric measurements according to the methods described previously [11]. Briefly, DCs were harvested and fixed by IC fixation buffer in the dark at room temperature for 30 min. Washed with permeabilization buffer and combined the recommended quantity of each antibody in an appropriate volume of permeabilization buffer. Staining was performed at room temperature for 30 min and cells were washed with flow cytometric staining buffer. Flow cytometry was performed using a FACSCalibur flow cytometer, and the data were analyzed using CellQuest software.

### *In vivo* splenic DCs or T cells flow cytometric analysis

To investigate the mechanism of LPS-induced DCs paralysis, the release of IFN-beta in splenic DCs and the expression of PD-1 or GITR in T cells in vivo was determined by Ag-pulsed DCs adoptive transfer combined with flow cytometric measurements according to the methods described previously [10]. Briefly, 5×10^5^ DCs (cultured for 4 d) pretreated with LPS (10 ng/ml), αIFN-beta (5 µg/ml) (MIB-5E9.1) blocking antibody prior to LPS stimulation were further conferred OVA peptide SIINFEKL pulse at the concentration of 2 µg/ml for 4 h. Then, the DCs were intraperitoneally transferred into C57BL/6 mice. 5 d after adoptive transfer, splenocytes of donors were prepared by deleting red blood cells and re-suspended in 400 µl buffer per 10^8^ total cells. The cells were further performed PD-1/GITR/CD4 or IFN-beta/CD11c antibodies staining. Then, the release of IFN-beta in splenic DCs and the expression of PD-1 or GITR expression in T cells were analyzed by flow cytometry.

### Ag-specific T cell proliferation assays

Antigen-specific proliferation assays were performed as described previously [11]. Briefly, DCs (cultured for 4 d) were pretreated with αIFN-beta (MIB-5E9.1) or αB7H1 (10F.9G2) neutralizing antibodies prior to LPS treatment. DCs treated with LPS, cytokines, IFN-beta was used as controls. Then, the DCs were conferred OVA peptide SIINFEKL (2 µg/ml) pulse and used as stimulator cells. Responder cells were prepared by the depletion of red blood cells from splenocytes of same background C57BL/6 mice. Stimulator cells were mixed with responders at a ratio of 1∶10 in 200 µl volume. After 5 d co-culture, Ag-specific T cell proliferation was determined via BrdU Cell Proliferation assays.

### Ag-specific IFN-gamma Elispot assays

To investigate the effect and mechanism of LPS on DCs-dependent specific CTL priming, Ag-specific IFN-gamma Elispot assays were performed using published methods [11]. Briefly, 5×105 DCs (cultured for 4 d) were pretreated with αIFN-beta (5 µg/ml) (MIB-5E9.1) prior to 12 h LPS treatment. DCs treated with LPS (10 ng/ml) was used as controls. Then, the DCs were further conferred 4 h OVA peptide SIINFEKL (2 µg/ml) pulse. After that, the DCs were intraperitoneally transferred into C57BL/6 mice. 5 d after adoptive transfer, splenocytes of donors were prepared and transferred into IFN–gamma antibody pre-coated plate (1×10^6^ cells per well). After that, the splenocytes were further re-stimulated with OVA peptide SIINFEKL at the final concentration of 2 µg/ml for 16∼20 h. Subsequently, splenocytes were washed away and the areas in which IFN–gamma had been bound were detected with combination of biotinylated anti-IFN–gamma detection antibodies and Streptavidinhorseradish peroxidase (Streptavidin-HRP). The substrate was added and the spots were counted. The Elispot data were presented as Spot Forming Units per million cells.

### RNA quantification and Quantitative real-time PCR analyses

Total RNA was extracted from DCs with TRI-zol reagent (Invitrogen) following the manufacturer’s instructions. RNA concentrations were determined with a NanoDrop instrument (NanoDrop Technologies). Reverse-transcription was performed to synthesize cDNA using PrimeScript Reverse Transcriptase. Quantitative real-time PCR (Q-PCR) analysis was performed as described previously [1]. The relative expression level of mRNAs was normalized by the level of beta-actin expression in each sample. The sequences of used primers are as following:

beta-actin: sense5′-CATCCGTAAAGACCTCTATGCCAAC-3′
antisense5′-ATGGAGCCACCGATCCACA-3′
IFN-beta: sense5′-CTGGCTTCCATCATGAACAA-3′
antisense5′-CATTTCCGAATGTTCGTCCT-3′
B7-H1: sense5′-AGAACGGGAGCTGGACCTGCTTGCGTTAG-3′
antisense5′-ATTGACTTTCAGCGTGATTCGCTTGTAG-3′
GITRL: sense5′-CTACGGCCAAGTGATTCCTG-3′
antisense5′-CCAGCATCTCGGGATACAAAG-3′
TGF-beta: sense5′- CTTCAATACGTCAGACATTCGGG −3′
antisense5′- GTAACGCCAGGAATTGTTGCTA-3′
IL-6: sense5′- GACAAAGCCAGAGTCCTTCAGAGAG-3′
antisense5′- CTAGGTTTGCCGAGTAGATCTC-3′
TNF-alpha: sense5′- CCACCACGCTCTTCTGTCTA-3′
antisense5′-TTTGCTACGACGTGGGCTAC-3′

### *In vivo* Treg generation determination

To determine the effects of LPS or IFN-beta on Treg generation *in vivo*, 5×10^5^ DCs (cultured for 4 d) were treated with LPS (10 ng/ml) or IFN-beta (10 ng/ml) for 12 h. Then, the DCs were conferred 4 h OVA peptide SIINFEKL pulse at the concentration of 2 µg/ml. After Ag pulse, the DCs were intraperitoneally transferred into C57BL/6 mice. 5 d after adoptive transfer, splenocytes and lymphocytes of lymph node were prepared by deleting red blood cells. Then, the cells were further performed intracellular Foxp3 and surface CD4, CD25 antibodies staining according to the methods described previously [20]. The proportion of Treg population was analyzed by flow cytometric measurements.

### Statistical analysis

All data were expressed as average of experimental data points, and standard error means were determined using the calculated standard deviation of a data set divided by the number of data points within the data set. Student’s t-test, one-way ANOVA with the Newman-Keulspost test was applied. Differences were considered significant at p<0.05.

## Results

### LPS treatment impairs nicotine-augmented DCs-mediated Ag-specific T cell proliferation and CTL priming

**Figure 1:**
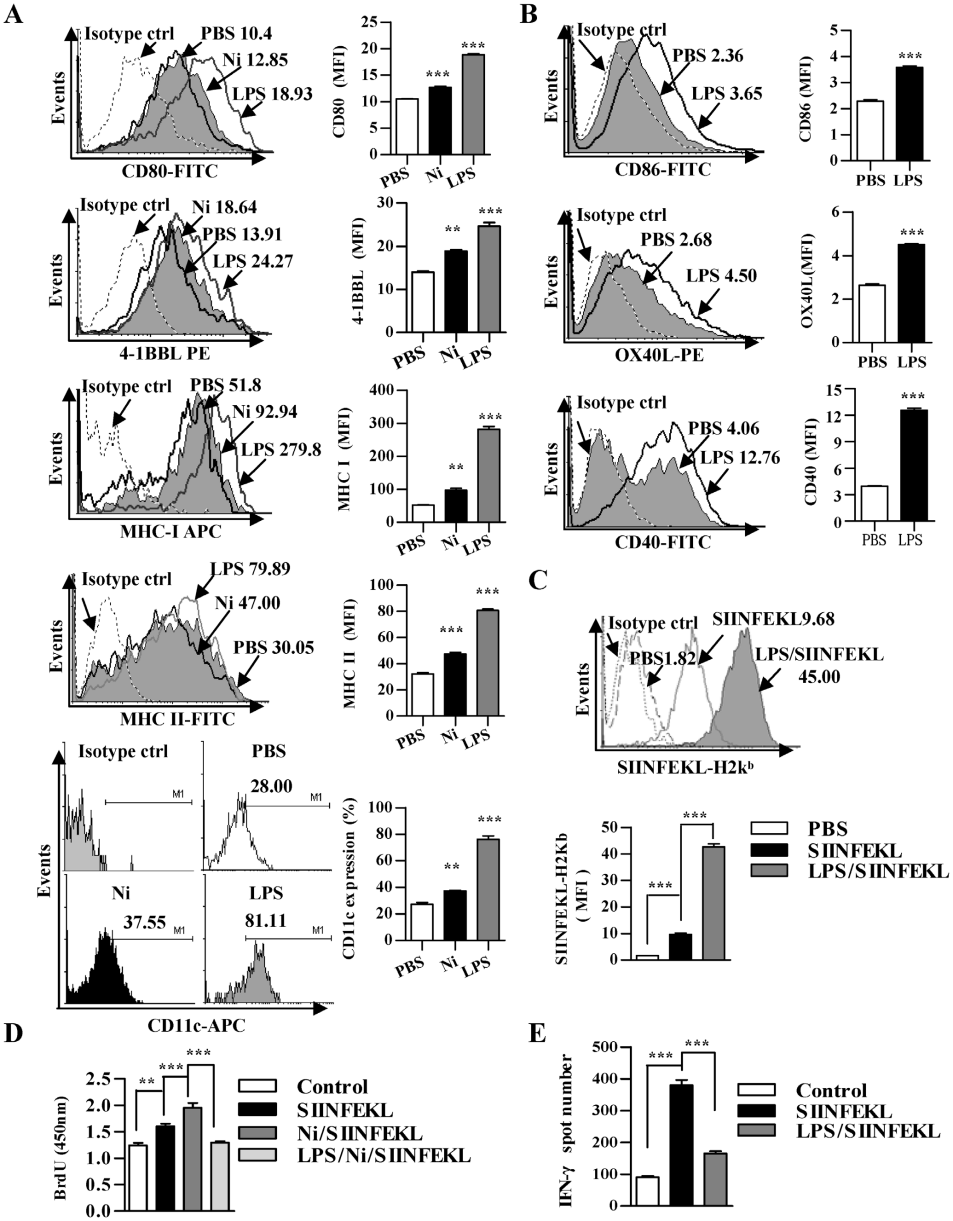
LPS treatment impairs nicotine-augmenting DCs-mediated Ag-specific T cell priming. DCs derived from murine bone marrow with GM-CSF and IL-4 were conferred with nicotine (10^−7^ M) or LPS (10 ng/ml) for 12 h. (A and B) the effect of nicotine or LPS on surface molecules expression was firstly determined via flow cytometry. After pulsed with OVA CD8^+^ T cell peptide SIINFEKL (2 µg/ml), the DCs were conferred flow cytometry assay to monitor SIINFEKL-H2Kb complex formation (C), co-incubation with splenocytes at a ratio of 1∶10 *in vitro* for 3 d (D) or intraperitoneal adoptive transfer to C57BL/6 donor (E). Then, the effect of nicotine or LPS on DCs-dependent T cell proliferation and Ag-specific CTL priming *in vivo* was determined via BrdU cell proliferation assay (D) and IFN-gamma Elispot assay (E), respectively. For IFN-gamma Elispot assay, the assay was performed 5 d after intraperitoneal transfer of 5×10^5^ DCs in the presence of SIINFEKL peptide (2 µg/ml) (n = 3). For flow cytometry (A-C), numbers in histogram indicated mean fluorescence intensity (MFI) of test samples except for CD11c flow cytometry assay indicated the positive percentages of analyzed samples. **p<0.01, ***p<0.001, one-way ANOVA with Newman-Keulspost test (A, C–E). ***p<0.001, Student’s *t* test (B). The data are presented as the mean ± SEM. One representative from 3 independent experiments is shown. Ni, nicotine.

Although our previous studies demonstrated that nicotine treatment clearly enhances DCs cross-presentation which has potential anti-tumor abilities [9]–[11], endotoxin tolerance in macrophages through ROS by increasing A20 expression was also demonstrated [18]. To investigate the effect of LPS on nicotine-augmented DCs-dependent T cell activation, DCs derived from bone marrow were treated with LPS and the effects of LPS on surface molecules expression, epitope-MHC I complex formation, T cell proliferation, Ag-specific CTL priming were explored. Consistent with previous reports [9]–[11], nicotine treatment clearly enhanced CD80, 4-1BBL, CD11c, MHC class I and II molecules expression up to 121%, 135%, 134%, 185% and 156%, respectively when compared with control cells (Figure 1A). Meanwhile, the treatment with LPS not only augmented these molecules expression with 179%, 176%, 289%, 537%, 265% up-regulation respectively (Fig. 1A) but also increased CD86, CD40 and OX40L expression (Figure 1B). Taking into account the vigorously pinocytosis of DCs (Figure S1), the effect of LPS on surface molecules’ up-regulation in DCs suggested that the treatment with LPS enhances co-stimulator molecules-mediated signaling of T cells priming.

To establish whether the treatment with LPS enhance antigen presentation, we pulsed DCs briefly (30 to 60 min) with antigen either concurrently with LPS or for the same length of time but without LPS exposure. We assessed antigen presentation on class I MHC molecules by detecting SIINFEKL-H2Kb, which is the complex of OVA derived CD8^+^ T epitope and MHC class I molecules. The treatment, which OVA peptide was firstly administered and at the end of antigen pulse, LPS was added, resulted in significantly Ag-presentation enhancement of the OVA peptide SIINFEKL when compared with OVA peptide administration without LPS (Figure 1C). We next determined the impact of LPS on T cells priming *in vitro*. Consistent with other reports [8], the level of DCs-dependent T cell proliferation (Figure 1D) and the release of IL-12 were obviously increased by the treatment with LPS (Figure S2). The pretreatment with LPS before antigen pulse obviously impaired nicotine-increased T cell proliferation (Figure 1D). Furthermore, when the presence of IFN-gamma in the cells was used to evaluate CTL priming, the treatment with LPS dramatically decreased the abilities of Ag-specific CTL priming *in vivo*, which reveals 57% inhibitory rate when compared with control DCs (Figure 1E). Taken together, these data suggested that LPS-mediated signaling drive the immunosuppressive program *in vivo*.

### High inducible expression of TGF-beta, TNF-alpha and IL-6 does not contribute to LPS induced DCs’ paralysis in DCs activated condition

**Figure 2:**
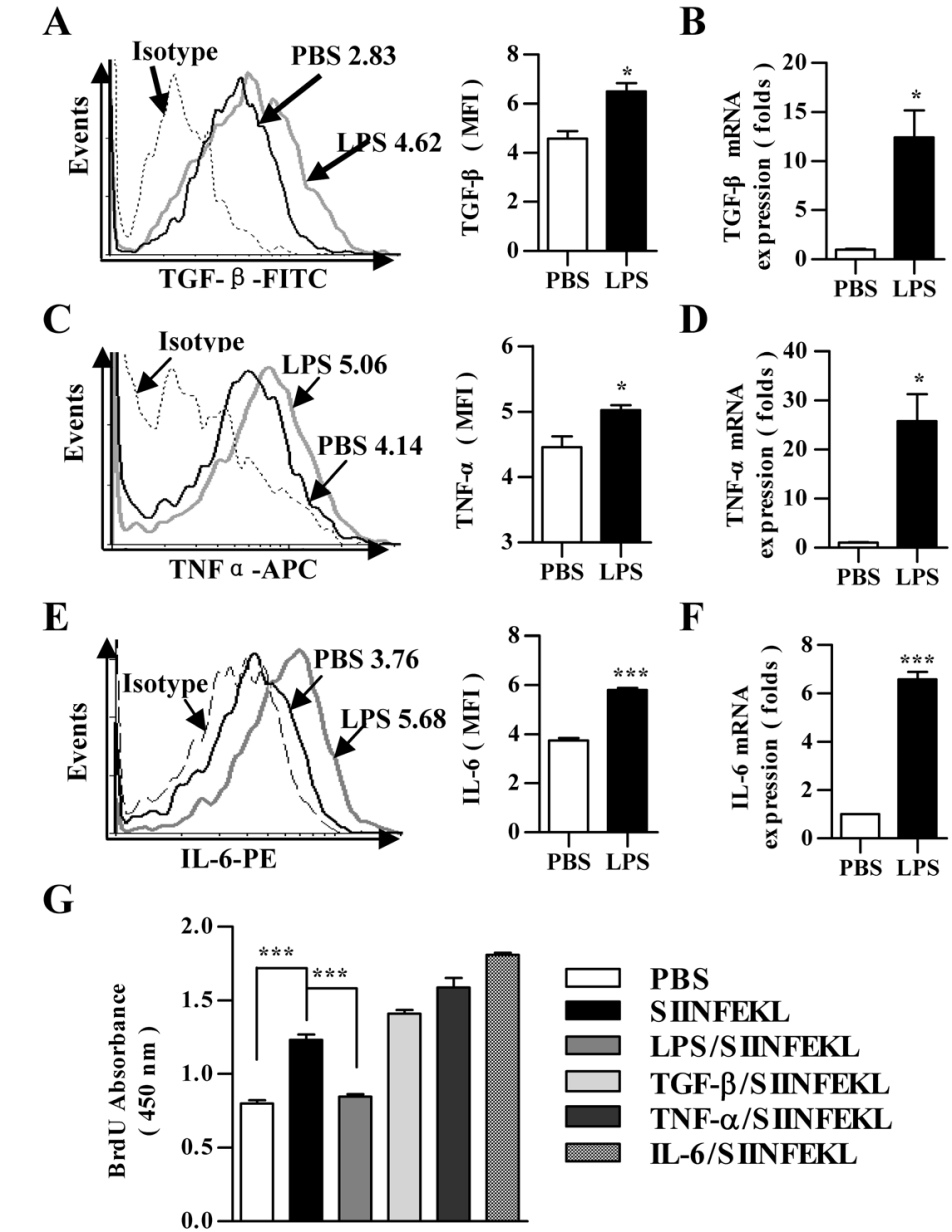
LPS-increased TGF-beta, TNF-alpha and IL-6 expression does not contribute to LPS-impaired DCs-dependent T cell proliferation. (A to F) DCs were treated with LPS (10 ng/ml) for 12 h. Then, the expression of TGF-beta (A and B), TNF-alpha (C and D) and IL-6 (E and F) was determined via flow cytometry (A, C and E) and real-time PCR analyses (B, D and F), respectively. For flow cytometry, numbers in histogram indicated MFI of analyzed samples. The data are presented as the mean ± SEM, n = 3. *p<0.05, ***p<0.001, Student’s *t* test. (G) Then, the effect of LPS, TGF-beta, TNF-alpha and IL-6 on T cell proliferation *in vitro* was assessed with BrdU cell proliferation assay by incubation LPS (10 ng/ml), TGF-beta (1 ng/ml), TNF-alpha (20 pg/ml) or IL-6 (20 pg/ml) treated and SIINFEKL (2 µg/ml) pulsed DCs with splenocytes at a ratio of 1∶10. The data are presented as the mean ± SEM, n = 6, ***p<0.001, one-way ANOVA with Newman-Keulspost test. One representative from 3 independent experiments is shown.

Immature DCs respond to pathogen-derived products by initiating a program of maturation that induce their migration to lymphoid organs and culminates in the up-regulation of MHC-peptide complexes, co-stimulatory molecules, and cytokines necessary for T cell activation [17], [20], [23]. In order to identify which cytokines orchestrate the immunosuppressive program during LPS exposure, the effects of LPS on the expression of TGF-beta, TNF-alpha, and IL-6 and the roles of these cytokines in LPS-induced impairment of DCs-dependent T cell proliferation were evaluated. The treatment with LPS clearly augmented TGF-beta expression in both protein and RNA level, which revealed 163% and 125% up-regulation, respectively (Figure 2A and 2B). The determination of TNF-alpha and IL-6 also derived the similar conclusion at the onset of LPS administration (Figure 2C to 2F); whereas, the expression of IL-10, IL-17A, IFN-alpha and IFNAR wanes with LPS exposure (Figure S3). To resolve the roles of increased cytokines in LPS-induced immunosuppressive program, we treated DCs with TGF-beta, TNF-alpha and IL-6, respectively. Despite the treatment with LPS revealed 31% inhibitory rate on Ag-specific T cell proliferation, the pretreatment with these cytokines had not affected DCs-dependent T cell proliferation anymore; whereas, Ag pulse obviously increased the abilities of DCs-dependent T cell priming (Figure 2G), indicating that other cytokines except for TGF-beta, TNF-alpha and IL-6 contribute to LPS-induced DCs’ impairment in DCs activated condition.

### Increased expression of IFN-beta contributes to LPS-induced immunosuppressive program of DCs

**Figure 3:**
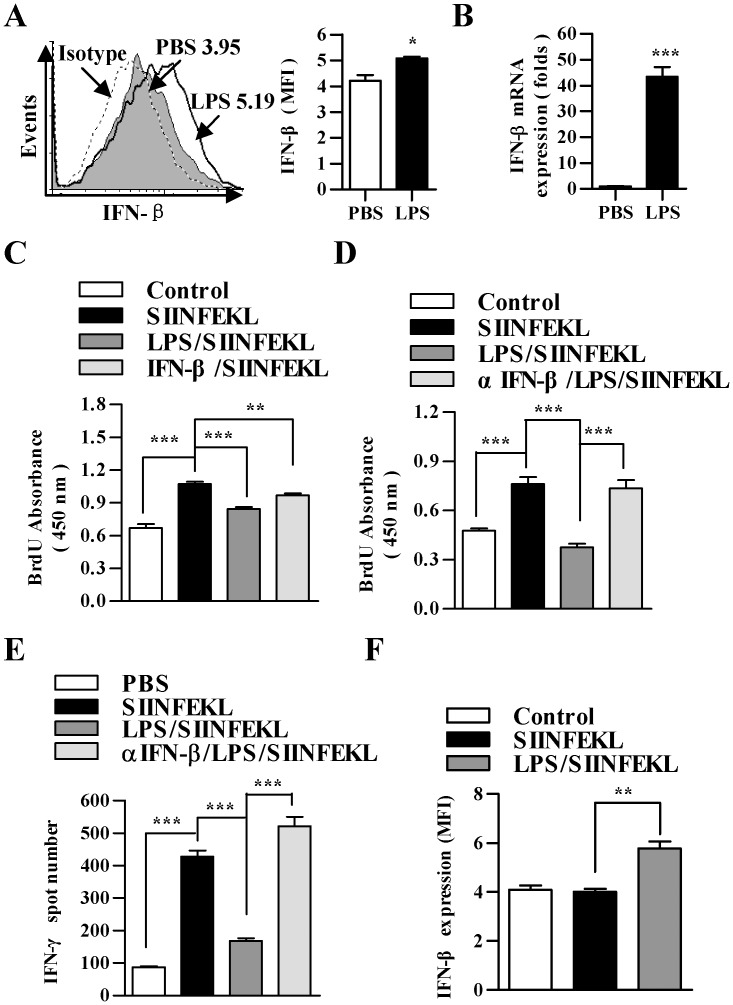
LPS-increased IFN-beta expression contributes to LPS-mediated DCs impairment. (A and B) DCs were treated with LPS (10 ng/ml) for 12 h and the expression of IFN-beta was determined via flow cytometry (A) and real-time PCR analyses (B), respectively. Numbers in histogram indicated MFI of analyzed samples. The data are presented as the mean ± SEM, n = 3. *p<0.05, **p<0.01, ***p<0.001, Student’s *t* test. (C to F) DCs were pretreated with αIFN-beta blocking antibody (5 µg/ml) (MIB-5E9.1) prior to 12 h LPS treatment (10 ng/ml) (D and E). DCs treated with LPS (10 ng/ml) or IFN-beta (10 ng/ml) were used as control. Then, the DCs were further conferred SIINFEKL pulse (2 µg/ml). After Ag pulse, the DCs were incubated with splenocytes at a ratio of 1∶10 (C and D) or intraperitoneally transferred to C57BL/6 mice (E and F) (5×10^5^ cells per mouse, n = 3). After 3 d incubation, T cell proliferation *in vitro* was assessed by BrdU cell proliferation assay (C and D). After 5 d adoptive transfer, Ag-specific CTL priming (E) and the release of IFN-beta in splenic DCs (F) *in vivo* was determined by IFN-gamma Elispot assay (E) and splenic DCs flow cytometry (F), respectively. The data are presented as the mean ± SEM, n = 6, **p<0.01, ***p<0.001, one-way ANOVA with Newman-Keulspost test.

IFN-I is critical for antiviral immunity; however, the blockade of IFN-I signaling restored lymphoid tissue architecture, and redirected the immune environment to enable control of infection [17]. To elucidate the potential cytokines which contribute to LPS-induced DCs immuosuppressive program, the effect of LPS on the release of IFN-beta and the role of IFN-beta in LPS-induced DCs’ impairment were evaluated. The treatment with LPS clearly enhanced IFN-beta expression in both protein and RNA level, (Figure 3A and B). The pretreatment with IFN-beta prior to antigen pulse revealed the similar inhibitory effect on T cell proliferation (Figure 3C). To resolve the role of IFN-beta in LPS-induced immunosuppressive program, we treated DCs with a αIFN-beta neutralizing antibody (MIB-5E9.1) prior to LPS stimulation. The blockade of IFN-beta efficiently diminished the effect of LPS on DCs-dependent T cell proliferation (Figure 3D), indicating the ability of IFN-beta blocking antibody to inhibit LPS-induced IFN-beta signaling *in vitro*. Analogous to the results of mixed lymphocyte reaction, the blockade of IFN-beta signaling with blocking antibody led to elevated Ag-specific CTL priming *in vivo* when compared with LPS treated DCs transfer (Figure 3E). Notably, the release of IFN-beta in splenic DCs rebounded when IFN-beta blocking antibody treatment was withdrawn (Figure 3F), indicating sensitive surveillance and rapid modulation of immunosuppressive state was completed through IFN-beta signaling.

### Increased B7H1/PD-1 signaling by IFN-beta contributes to LPS-induced DCs paralysis

**Figure 4:**
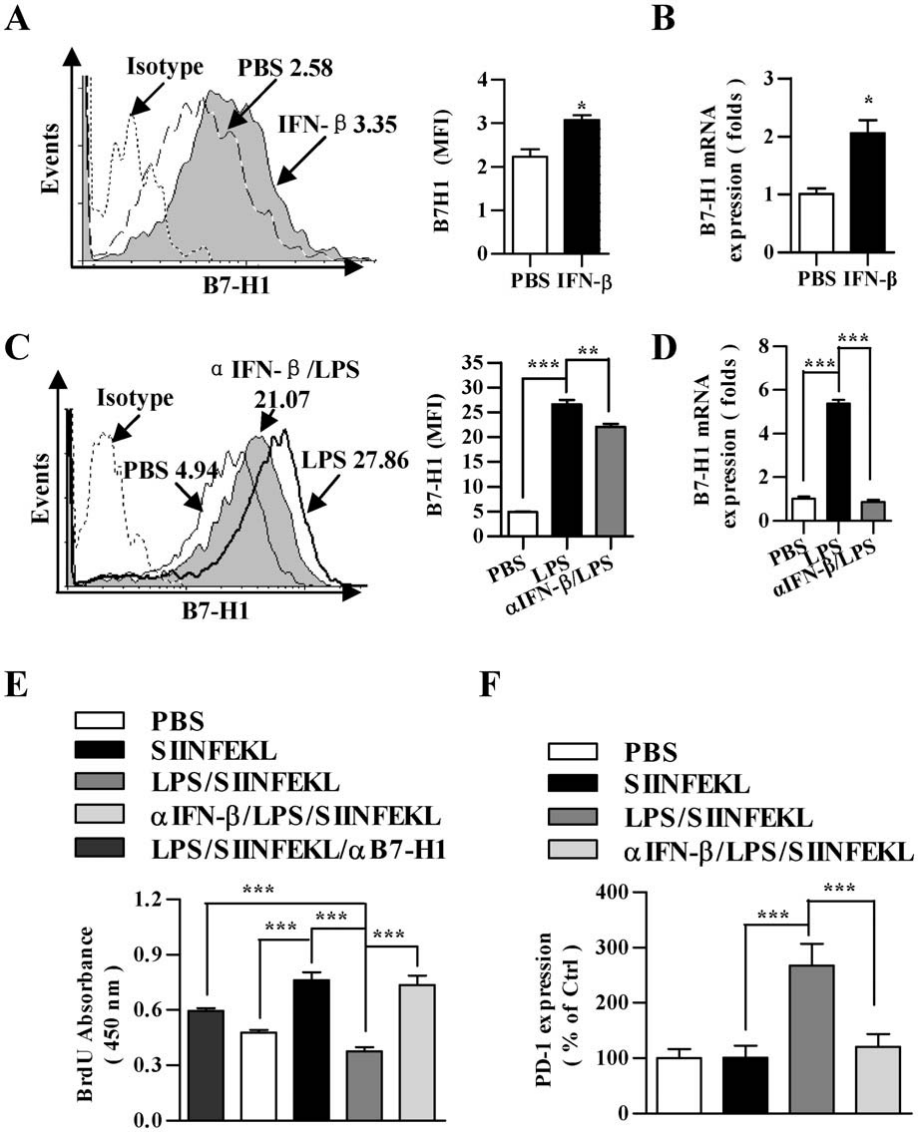
IFN-beta enhanced B7H1/PD-1 signaling contributes to LPS impaired T cell proliferation. (A and B) DCs were treated with IFN-beta (10 ng/ml) for 12 h and the expression of B7H1 was determined by flow cytometry (A) and real-time PCR (B), respectively. (C and D) DCs were pretreated with αIFN-beta blocking antibody (5 µg/ml) (MIB-5E9.1) or PBS 2 h prior to 12 h LPS (10 ng/ml) stimulation and the effect of αIFN-beta blocking antibody on LPS-increased B7H1 expression was determined via flow cytometry (C) and real-time PCR analyses (D), respectively. Numbers in histogram (A and C) indicated MFI of analyzed samples. The data are presented as the mean ± SEM, n = 3. To investigate the roles of LPS-increased IFN-beta and B7H1 in LPS-induced DCs’ dysfunction, αB7H1 blocking antibody (5 µg/ml) (10F.9G2) or αIFN-beta blocking antibody (5 µg/ml) (MIB-5E9.1) (E and F) were used prior to LPS treatment. After that, the DCs were further conferred SIINFEKL (2 µg/ml) pulse. Then, these DCs were incubated with splenocytes at a ratio of 1∶10 *in vitro* (E) or intraperitoneally transferred to C57BL/6 mice (F) (5×10^5^ cells per mouse, n = 3). After 3 d incubation, T cell proliferation *in vitro* was assessed by BrdU cell proliferation assay (E). After 5 d adoptive transfer, the expression of PD-1 in splenic T cells (F) was determined by flow cytometry (F). The data are presented as the mean ± SEM, n = 6, *p<0.05, **p<0.01, ***p<0.001, student’s *t* test or one-way ANOVA with Newman-Keulspost test.

It was reported that programmed cell death 1 ligand 1 (PD-L1, B7H1), a 40 kDa type 1 transmembrane protein, could increase antigen cross-presentation but impair cross-priming [23] and contribute to TGF-beta mediated Treg priming [20]. In order to identify the mechanism orchestrating the immunosuppressive program mediated by increased IFN-beta by treatment with LPS, we next sought to understand the effect of LPS on B7H1 expression and the role of B7H1 signaling in LPS-impaired T cell priming. Importantly, the treatment with IFN-beta significantly increased the expression of B7H1 in DCs when compared with untreated cells (Figure 4A and B). This up-regulation of B7H1 strictly correlated to LPS treatment, as the treatment with LPS also showed a strongly enhanced expression of B7H1 in DCs; whereas, the blockade of IFN-beta signaling with neutralizing antibody abrogated the effect of LPS on B7H1 expression (Figure 4C and D). Notably, in contrast to the strong inhibitory effect of LPS, the blockades of both IFN-beta and B7H1 signaling with αIFN-beta (MIB-5E9.1) or αB7H1 (10F.9G2) neutralizing antibodies efficiently abrogated the effect of LPS on DCs-dependent T cell proliferation (Figure 4E).

To confirm whether LPS-mediated impairment of DCs priming capacity was due to enhanced expression of B7H1, we analyzed the expression of PD-1 in splenic T cells of DCs adoptive transferred donors. In contrast to LPS treatment, which caused a strong up-regulation of PD-1, the pretreatment with αIFN-beta neutralizing antibody (MIB-5E9.1) even more efficiently reversed the effect of LPS on PD-1 expression (Figure 4F). These results indicated that the impaired T cell activation observed in co-cultures with LPS-treated DCs was mediated by the up-regulation of B7H1 and PD-1.

### LPS induces Foxp3^+^TREG priming *in vivo* by enhancing GITRL signaling

**Figure 5:**
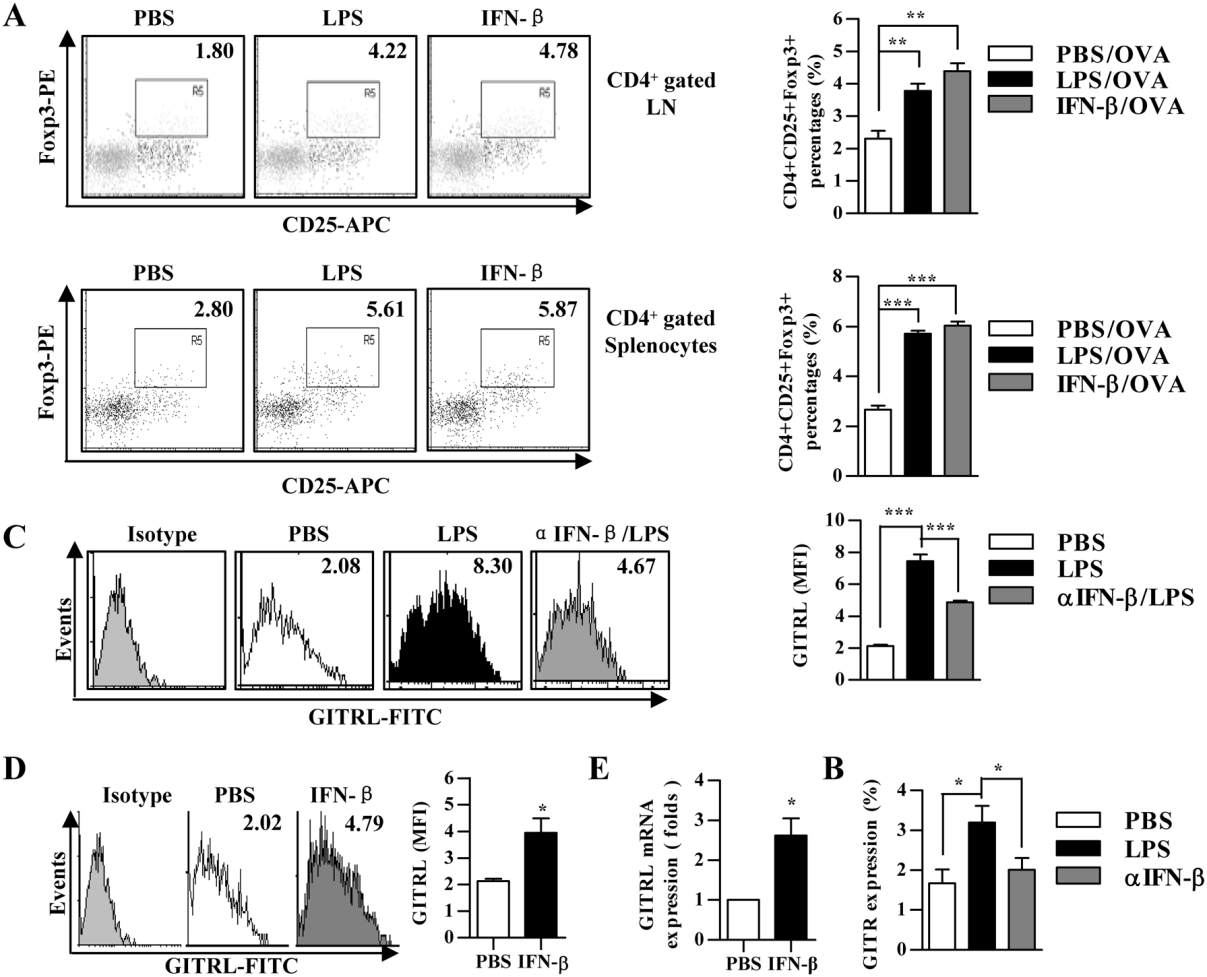
IFN-beta induced Foxp3^+^Treg priming contributes to LPS mediated DCs impairment. (A and E) 5×10^5^ DCs were treated with IFN-beta (10 ng/ml) or LPS (10 ng/ml) for 12 h (A) or pretreated with αIFN-beta blocking antibody (5 µg/ml) (MIB-5E9.1) prior to 12 h LPS treatment (10 ng/ml) (E), which were further conferred SIINFEKL (2 µg/ml) pulse. After that, the DCs were intraperitoneally transferred to C57BL/6 mice (n = 3). 5 d after adoptive transfer, the proportion of CD4^+^CD25^+^Foxp3^+^ Treg *in vivo* (A) and the expression of GITR in splenic T cells (E) were determined by flow cytomentry. Numbers in dot plot indicated positive cells’ percentages of gated cells. The data are presented as the mean ± SEM, n = 3, **p<0.01, ***p<0.001, one-way ANOVA with Newman-Keulspost test. (B to D) DCs were treated with αIFN-beta blocking antibody (5 µg/ml) (MIB-5E9.1) prior to LPS (10 ng/ml) stimulation or IFN- beta (10 ng/ml). The expression of GITRL was determined via flow cytometry (B and C) and real-time PCR (D), respectively. Numbers in histogram indicate MFI of each DCs population. The data are presented as the mean ± SEM, n = 3, *p<0.05, **p<0.01, ***p<0.001, one-way ANOVA with Newman-Keulspost test. LN, lymph node.

Treg, a subpopulation of T cells which maintain tolerance to self-antigens, could be induced by the treatment of IFN-beta through the up-regulation of GITRL in DCs [22]. To investigate whether LPS-mediated impairment of DCs priming capacity was due to increased Treg population, we analyzed *in vivo* Treg priming by the treatment with LPS. Analogous to IFN-beta treatment, the treatment with LPS led to elevated Treg priming capacity when compared with untreated DCs, which increased from 1.80 to 4.22% and 2.80 to 5.61% in lymph node and spleen, respectively (Figure 5A); whereas the treatment with IFN-beta augmented Treg priming capacity to 191% and 227% in lymph node and spleen, respectively (Figure 5A).

To investigate whether LPS-mediated Treg priming was due to enhanced expression of GITRL, we analyzed the effect of LPS and IFN-beta on the expression of GITRL *in vitro*. In contrast to LPS treatment, which had strong expression of GITRL, the pretreatment with αIFN-beta neutralizing antibody (MIB-5E9.1) efficiently decreased the effect of LPS on GITRL expression (Figure 5B). Similar to LPS treatment, the treatment with IFN-β also achieved enhanced GITRL expression in both protein and RNA levels (Figure 5C and D). These results indicated that LPS-induced Treg priming has closely correlated to GITRL up-regulation. Next, we co-cultured Ag-pulsed DCs with naïve T cells in vitro. Enhanced Treg priming by LPS or TGF-beta treatment was achieved when compared with untreated DCs, which revealed an increased proportion of CD4+Foxp3+ T cells (Figure S4A). As Foxp3^+^ T cell also had higher GITR expression (Figure S4B), these results indicated that GITRL signaling play potential role in LPS-induced Treg priming. To further prove that enhanced Treg priming after the treatment with LPS was due to the interaction of GITRL expressed by DCs with GITR on the surface of naïve T cells, we performed *in vivo* Treg priming experiment in the presence of αIFN-beta blocking antibody (MIB-5E9.1). Indeed, the enhanced GITR expression in splenic T cells was overcome in the presence of αIFN-beta blocking antibody, indicating that the effect of LPS on Treg priming dependent on the interaction of GITRL on DCs with GITR on T cells (Figure 5E).

### LPS up-regulated IFN-beta expression by activating NF-kappaB pathway

**Figure 6:**
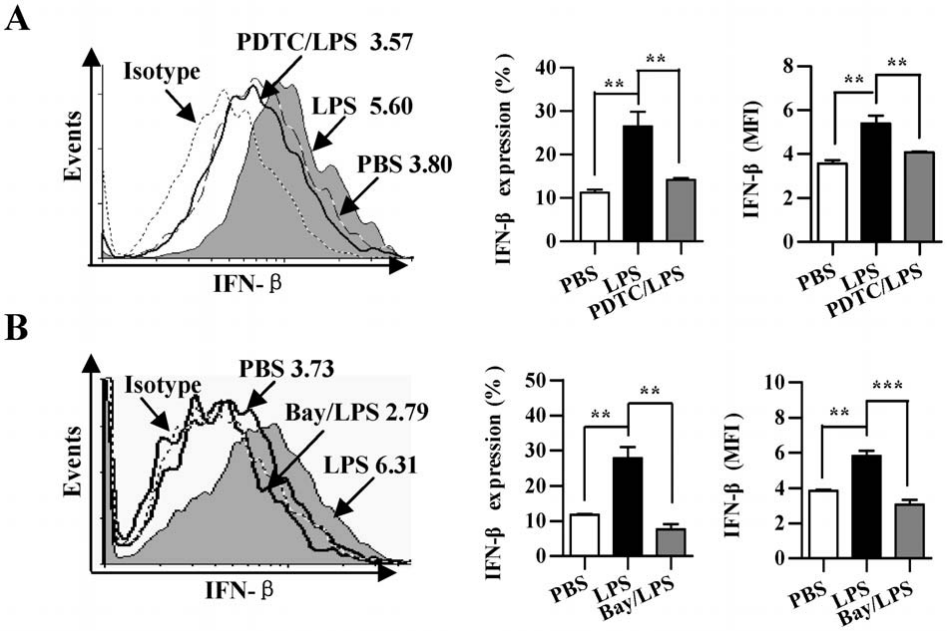
LPS up-regulated IFN-beta expression by activating NF-kappaB pathway. (A and B) DCs were pretreated with the NF-kappaB kinase inhibitor 40 µM PDTC (A) or 20 µM Bay 11-7082 (B) 2 h prior to LPS (10 ng/ml) 12 h stimulation. The expression of IFN-beta was determined by flow cytometry. Numbers in histogram indicate MFI of analyzed population (left). Statistical analysis of IFN-beta positive percentages (middle) and MFI (right) was shown. The data are presented as the mean ± SEM, n = 3, **p<0.01, ***p<0.001, one-way ANOVA with Newman-Keulspost test. One representative from 3 independent experiments is shown.

Pauls E *et al* studies showed that IKKbeta play essential role in production of type I interferons by plasmacytoid dendritic cells [24]. Finally, we investigated the role of NF-kappaB activation in LPS augmented IFN-beta expression. To this end, DCs were treated with the NF-kappaB inhibitor PDTC and Bay 11-7082 to inhibit related kinases activities before they conferred LPS treatment. After 16 h, DCs were collected and analyzed for IFN-beta expression. Confirming the results obtained by other groups [25], the treatment with LPS revealed an increase in the proportion of IFN-beta expressing DCs (Figure 6). The usage of the PDTC and Bay 11-7082 obviously abrogated the effect of LPS on IFN-beta expression when compared with LPS-treated DCs, which achieved 46.5% and 72.4% inhibitory rate, respectively (Figure 6). These results demonstrated that LPS up-regulates IFN-beta expression by activating NF-kappaB pathway.

## Discussion

**Figure 7:**
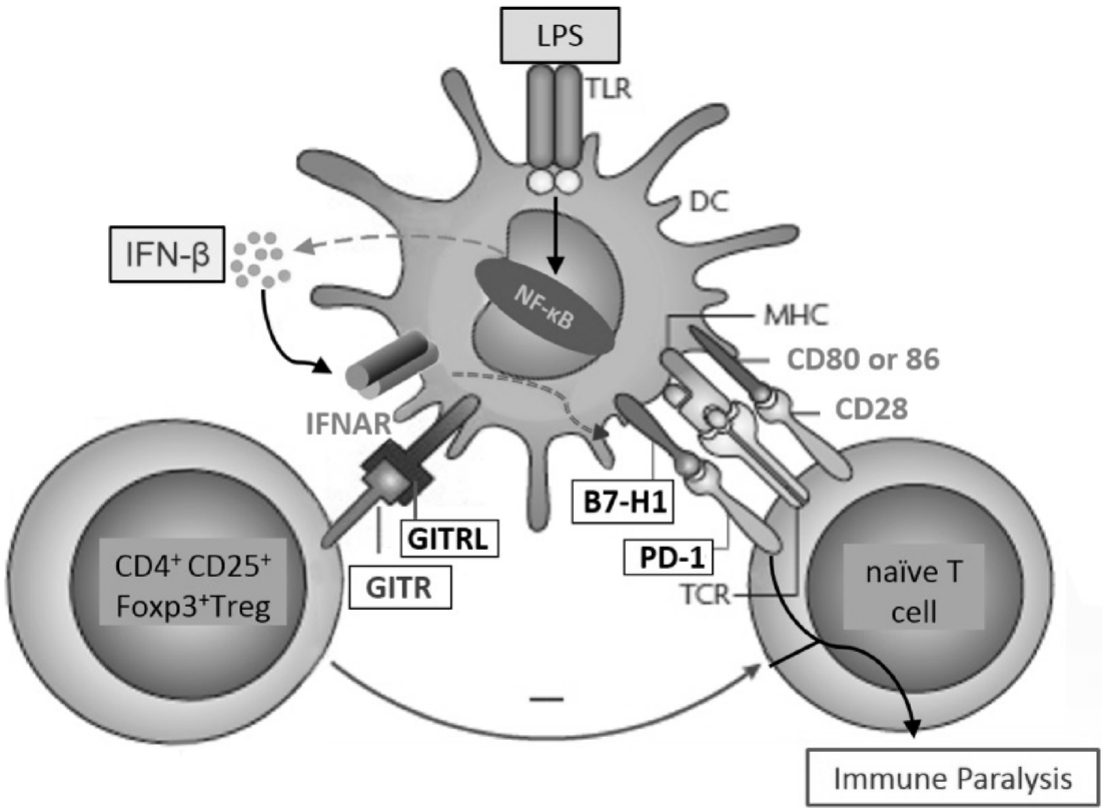
Model of IFN-beta mediating LPS-induced DCs immune paralysis. IFN-beta, which is increased by LPS-induced NF-kappaB activation, leads to increased B7H1 and GITRL expression in DCs. Then, increased interactions of B7H1/PD-1 combined with GITRL/GITR interaction dependent Treg priming impair DCs-dependent T cell activation.

In this study, we investigated the effect of LPS treatment in DCs on nicotine-enhanced Ag-presentation of soluble OVA and on Ag-specific T cells priming. We demonstrated that LPS treatment increases the release of IFN-beta both *in vitro* and *in vivo*. Concurrently, IFN-beta strongly enhanced the expressions of the co-inhibitory molecules B7H1 and GITRL in DCs. Increased Ag-presentation combined with enhanced co-inhibitory signaling or increased Treg proportion resulted in impaired activation of naïve OVA-specific T cells (Figure 7).

Immune paralysis refers to DCs failing to mount adaptive immunity towards secondary microbial infections [15], [26]. Although systemic dissemination of bacteria initiated T cell paralysis which was mediated by the release of IFN-I from splenic macrophages was reported [14], the potential mechanisms of IFN-I mediating DCs paralysis in autocrine or paracrine manner have never been fully understood. Immature DCs respond to pathogen-derived products by initiating a program of maturation, which culminates in the up-regulation of MHC-peptide complexes. In the present studies, both nicotine and LPS treatment obviously increase MHC class I and II molecules expression (Figure 1A), indicating that LPS-induced DCs’ paralysis is not due to the low expression of MHC molecules. Here, we demonstrated that LPS impairing DCs-dependent T cells priming was due to the up-regulation of IFN-beta, which concurrently enhances the expressions of the co-inhibitory molecules B7H1 combined with increased Ag-specific Treg priming. Further studies revealed that Ag-specific Treg priming is LPS-augmented GITRL expression dependent. Our results indicate that IFN-beta might be potential therapeutic molecules to overcome LPS-induced DCs paralysis.

IFN-I, a pleiotrophic inflammatory cytokine, was reported to increase CTL immunity by enhancing DCs maturation [27] and promoting antigen presentation [28]. Meanwhile, the inhibitory effect of IFN-I on T cells priming, in particular IFN-beta, has also been documented [16], [29], [30]. Considering that LPS clearly enhanced IFN-beta expression (Figure 3A and B) and had no effect on IFN-alpha expression (), the paradoxical conclusion might be due to the types of IFN used in these experiments. Li Y *et al*’s studies also derived the similar conclusions [18], [31]. B7H1, a 40 kDa type I transmembrane protein, play a major role in suppressing the immune system. The interactions of B7H1 and PD-1 not only regulates the generation of adaptive Treg [19] but also causes an impaired cross-priming of naive OVA-specific CD8^+^ T cells [16], [17], [23]. It’s our interest to explore whether B7H1 contribute to LPS-induced IFN-beta-mediated immune paralysis. To our surprise, both LPS and IFN-beta treatment clearly augmented B7H1 expression. An efficient recovery of LPS-induced paralysis *in vitro* (Figure 4E) and decreased PD-1 expression in splenic T cells *in vivo* (Figure 4F) by the blockade of B7H1 or IFN-beta indicates that the interaction between B7H1 and PD-1 is involving in LPS-induced immune paralysis. Apart from B7H1, other co-inhibitory molecules, such as B7DC, B7H3, and B7-H4 were also reported to be involved in T cell immune suppression [32]–[34]. In the present study, a partial recovery of T cell proliferation by the blockade of B7H1 was achieved when compared with IFN-beta blocking treatment (Figure 4E), indicating that other co-inhibitory molecules might take part in LPS-induced DCs paralysis and further studies are needed.

Apart from IFN-I, we also mentioned the overlapping roles of IL-10 and IFN-I in impairing CTL immunity of sepsis patients [13], [14], [27]. Meanwhile, IL-6 [35] and TNF-alpha [36] were also reported to be involved in the impairment of T cells priming. In the present study, the treatment with LPS having no effect on the expression of IL-10 and IL-17A exclude the possible roles of these cytokines in LPS-induced paralysis (Figure S3). Despite the release of IL-6 and TNF-alpha was enhanced by the treatment with LPS, the treatment with these cytokines did not reveal the similar inhibitory effect when compared with LPS treatment (Figure 2G). This may be due to the maturation status of DCs used in our experiment, as the DCs in our system was derived from bone marrow with GM-CSF and IL-4 and cultured for 4 d, which is definitely recognized as relative immature DCs [11]. Despite TGF-beta could be up-regulated by the treatment with LPS and IFN-beta (Figure S4C), which concomititantly increased Ag-specific Treg generation (Figure S4), the treatment with TGF-beta could not inhibit Ag-specific T cell proliferation anymore (Figure 2G). Interestingly, we found that the treatment with subdose Ag (100 ng/ml OVA) facilitates Treg priming (Figure S4); whereas normal dose Ag stimulation (2 µg/ml OVA peptide) increases T cell proliferation (Figure 2G), indicating that the treatment with Ag dose would be an important factor to affect DCs-dependent T cells activation or inhibition. Kretschmer, K *et al*’s studies also revealed that subdose antigen stimulation facilitated Ag-specific Treg inducing and expanding in the presence of TGF-beta [37]. Hence, the failure of TGF-beta in inhibiting T cell proliferation might be due to the insufficient Treg priming in mixed lymphocytes reaction.

By using a soluble form of GITR, Grohmann U demonstrated that type I IFNs are required for pDCs to possess reverse signaling through GITRL and result in the onset of immune regulation [38], indicating that reverse signaling of GITRL has a tolerogenic effect on DCs. Our present studies provide evidences that the treatment with IFN-beta enhanced GITRL expression (Figure 5B and C) and driven differentiation of DCs towards a tolerogenic phenotype (Figure 3C). Our previous studies revealed that TGF-beta present in the microenvironment of lung cancer increase Treg generation by upregulating GITRL expression [20]. IFN-beta facilitated Treg proliferation and accumulation by upregulation of GITRL in DCs was also documented [22]. Therefore, GITRL expression on DCs might have two concurrent effects: GITRL triggering on maturation of tolerogenic DCs and GITR triggering on Treg expansion. In the present study, the treatment with LPS or IFN-beta not only enhanced GITRL expression (Figure 5B and C) but also promoted the release of TGF-beta (Figure S5C). Hence, it is no surprise to find that Ag-specific Treg proportion in DCs adoptive transferred donors was increased by the treatment with LPS or IFN-beta (Figure 5A).

GITR was originally discovered as a gene upregulated in dexamethasone-treated murine T cell hybridomas [39]. In contrast to low basal expression on naïve murine CD4^+^ and CD8^+^ T cell, and very low expression on human T cells, Treg cells and NK cells constitutively express high levels of GITR [40]. By using transgenic expression of GITRL, GITR triggering was found to regulate the balance between regulatory and effector CD4^+^ T cells by enhancing proliferation of both populations in parallel [41]. Adoptively-transferred Treg from tumor-bearing animals lose Foxp3 expression when treated with DTA-1, whereas Treg from naïve mice maintain Foxp3 expression [42]. Hence, despite the GITR agonist antibody DTA-1 could overcome self-tolerance and reverse Treg suppression, secondary melanoma challenge tumors cannot be rejected during the early stages of primary tumor growth, unless Treg are depleted [40], indicating that the clinical consequence of an immune response might even depend on the ratio of effector vs regulatory T cells. In addition to species specific of GITRL/GITR binding [43], GITR is not triggered by GITRL monomer and dimer and that the level of stimulation increases with oligomerization [44]. Whether the treatment of LPS or IFN-beta affects GLTRL oligomerization awaits further investigation.

Although DCs do not have a unique cell surface phenotyoe, a constellation of cell surface molecules, including CD11c, CD83 and MHC class II molecules, has enabled investigators to demonstrate their function *in vivo* and *in vitro*. In this study, murine bone marrow derived 4 d DCs without LPS maturation were used to investigate the mechanism of LPS-induced paralysis. Despite nicotine augmented 1.6 and 1.3 folds expression of CD11c and MHC class II, LPS treatment increased about 2.7 and 2.8 folds up-regulation of these molecules, which are in line with previous reports [12], indicating that DCs used in our studies is relatively immature DCs. Apart from immune cells, endothelial cell, fibroblast and other cells were also reported to express IFN-beta and other cytokines in LPS stimulated condition [45]–[47]. Hence, despite our studies demonstrated that IFN-beta contributes to LPS-induced DCs paralysis, it cannot exclude the possibilities that IFN-beta and other cytokines expressed by other cells facilitate pathogen-induced DCs’ paralysis, which awaits further investigation.

Taken together, our data provide a new molecular mechanism for LPS-induced T cell tolerance, which is mediated by the combined action of increased Treg proportion and enhanced signaling via the co-inhibitory molecules B7H1 and GITRL (Figure 7). This mechanism provides new insights into the molecular mechanisms of T cell inactivation and might thus open new opportunities for therapeutic intervention in T cell-mediated autoimmune diseases and persistent infection.

## Supporting Information

Figure S1 **LPS treatment increase DCs’ pinocytosis.** DCs derived from bone marrow were treated with LPS (10 ng/ml) for 12 h and further pulsed with lucifer yellow for 30 min. Then, the DCs’ pincytosis was determined by flow cytometry.
(TIF)

Figure S2 **LPS treatment increase IL-12 release.** DCs treated with LPS (10 ng/ml) for 12 h were further conferred 4 h SIINFEKL pulse (2 µg/ml). Then, these DCs were incubated with splenocytes at a ratio of 1∶10 *in vitro*. After 5 d incubation, IL-12 and IL-4 release of suspension were determined by ELISA. Data were given as mean ± SEM, n = 3, **p<0.01, ***p<0.001, one-way ANOVA with post Newman-Keuls test. A representative out of 3 independent experiments was shown.
(TIF)

Figure S3 **LPS stimulation has no effect on IL-10, IL-17A, IFN-α and IFNAR expression in DCs.** (A to D) DCs were treated with LPS (10 ng/ml) for 12 h. 1XBFA was added 6 h prior to the experiment point. The expression of IL-10 (A), IL-17A (B), IFN-α (C) and IFNAR (D) was determined by flow cytometry. Data were given as mean ± SEM, n = 3. A representative out of 3 independent experiments was shown.
(TIF)

Figure S4 **Both LPS and TGF-β increase Treg priming** ***in vitro*** **.** (A and B) DCs were treated with LPS (10 ng/ml) or TGF-beta (5 ng/ml) for 12 h which were further conferred OVA pulse at the final concentration of 100 ng/ml. Then, the DCs were coincubated with splenocytes at the ratio of 1∶10. After 3 d co-culture, CD4^+^Foxp3^+^ and CD4^+^GITR^+^Foxp3^+^T cell proportion was analyzed by flow cytomentry. Numbers in histogram indicated the positive cells percentages of each analyzed population. Data were given as mean ± SEM, n = 3. **p<0.01, ***p<0.001, one-way ANOVA with post Newman-Keuls test. A representative out of 3 independent experiments was shown. (C) IFN-beta up-regulates TGF-beta expression in DCs. DCs were treated with LPS (10 ng/ml) or IFN-beta (10 ng/ml) for 12 h and the expression of TGF-beta was determined by flow cytometry. Data were given as mean ± SEM, n = 3. A representative out of 3 independent experiments was shown.
(TIF)
