# Correction: Increased Antigen Presentation but Impaired T Cells Priming after Upregulation of Interferon-Beta Induced by Lipopolysaccharides Is Mediated by Upregulation of B7H1 and GITRL

**DOI:** 10.1371/journal.pone.0108120

**Published:** 2014-09-10

**Authors:** The PLOS ONE Staff

## Notice of Republication

This article was republished on August 27th, 2014, to correct an error where Figure 1 was posted as a duplicate of Figure 2. Please download the PDF again to view the corrected article. The originally published, uncorrected article and the republished, corrected article are provided here for reference.

## Supporting Information

File S1 Originally published, uncorrected article
(PDF)

File S2 Republished, corrected article
(PDF)
