# Supplementary figures and images for: Increased Antigen Presentation but Impaired T Cells Priming after Upregulation of Interferon-Beta Induced by Lipopolysaccharides Is Mediated by Upregulation of B7H1 and GITRL
Source: PLoS One. 2014 Aug 21;9(8):e105636. doi: 10.1371/journal.pone.0105636 (PMC4140801; doi:10.1371/journal.pone.0105636)

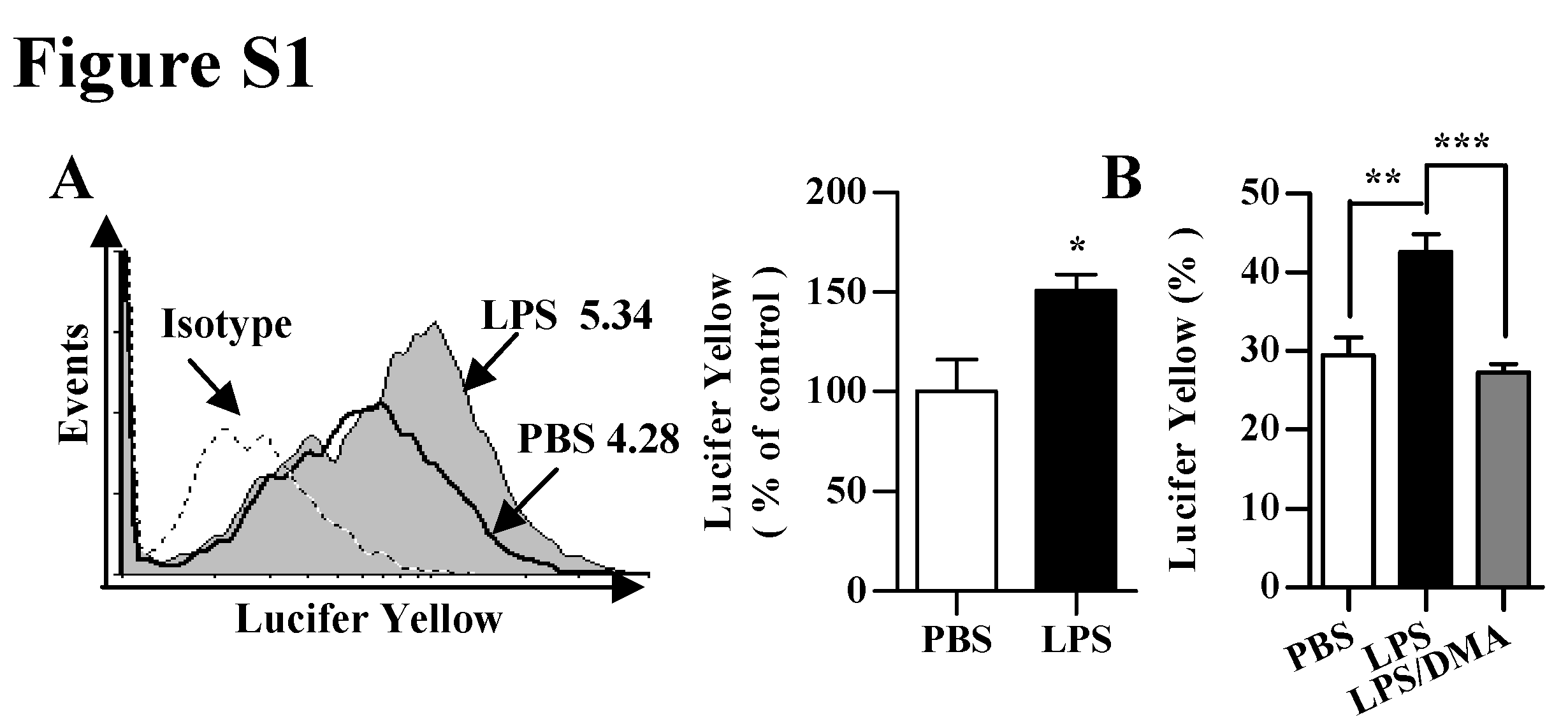

Supplement: Figure S1 — LPS treatment increase DCs’ pinocytosis. DCs derived from bone marrow were treated with LPS (10 ng/ml) for 12 h and further pulsed with lucifer yellow for 30 min. Then, the DCs’ pincytosis was determined by flow cytometry. (TIF) [file pone.0105636.s001.tif]

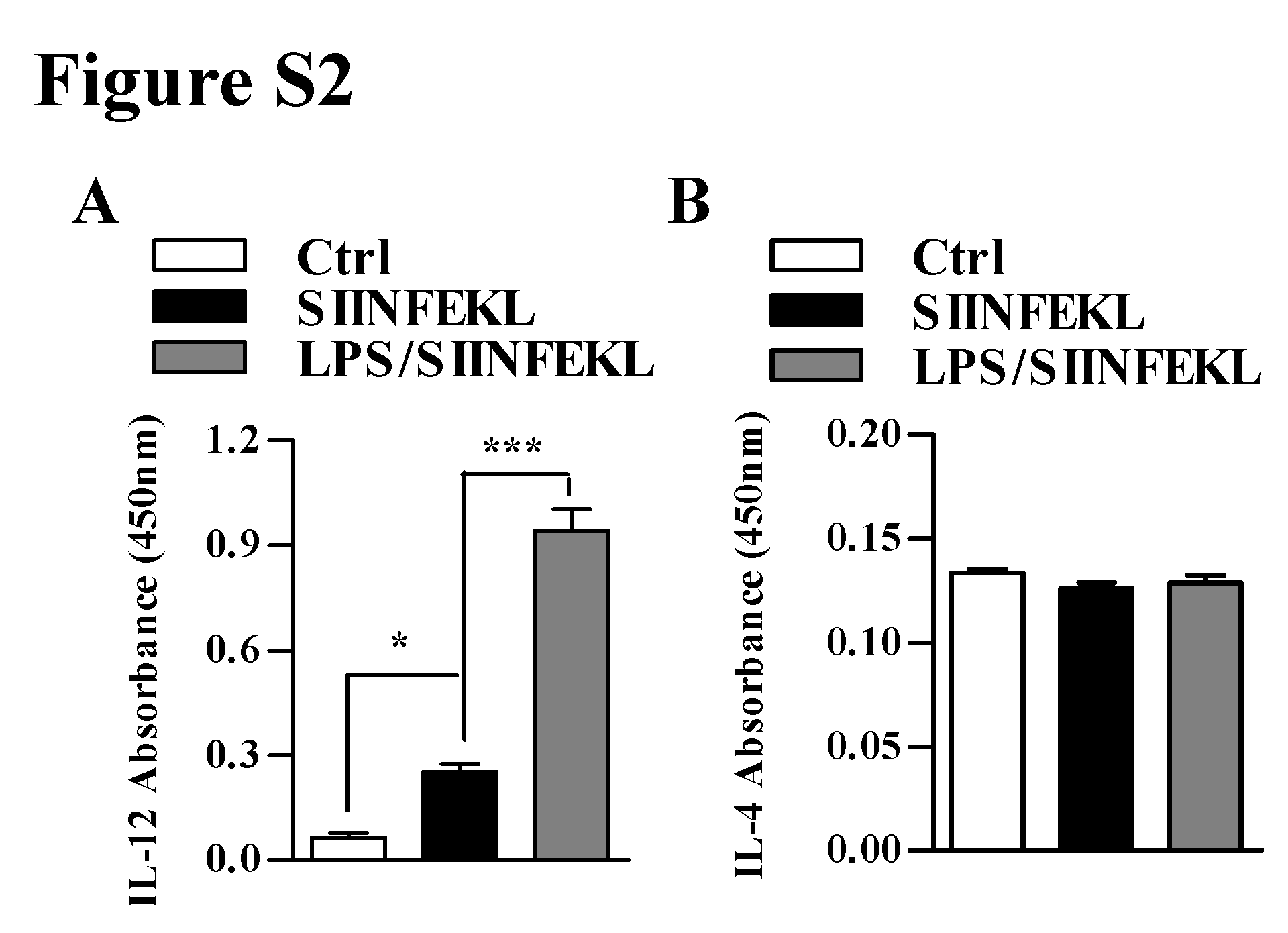

Supplement: Figure S2 — LPS treatment increase IL-12 release. DCs treated with LPS (10 ng/ml) for 12 h were further conferred 4 h SIINFEKL pulse (2 µg/ml). Then, these DCs were incubated with splenocytes at a ratio of 1∶10 in vitro. After 5 d incubation, IL-12 and IL-4 release of suspension were determined by ELISA. Data were given as mean ± SEM, n = 3, **p<0.01, ***p<0.001, one-way ANOVA with post Newman-Keuls test. A representative out of 3 independent experiments was shown. (TIF) [file pone.0105636.s002.tif]

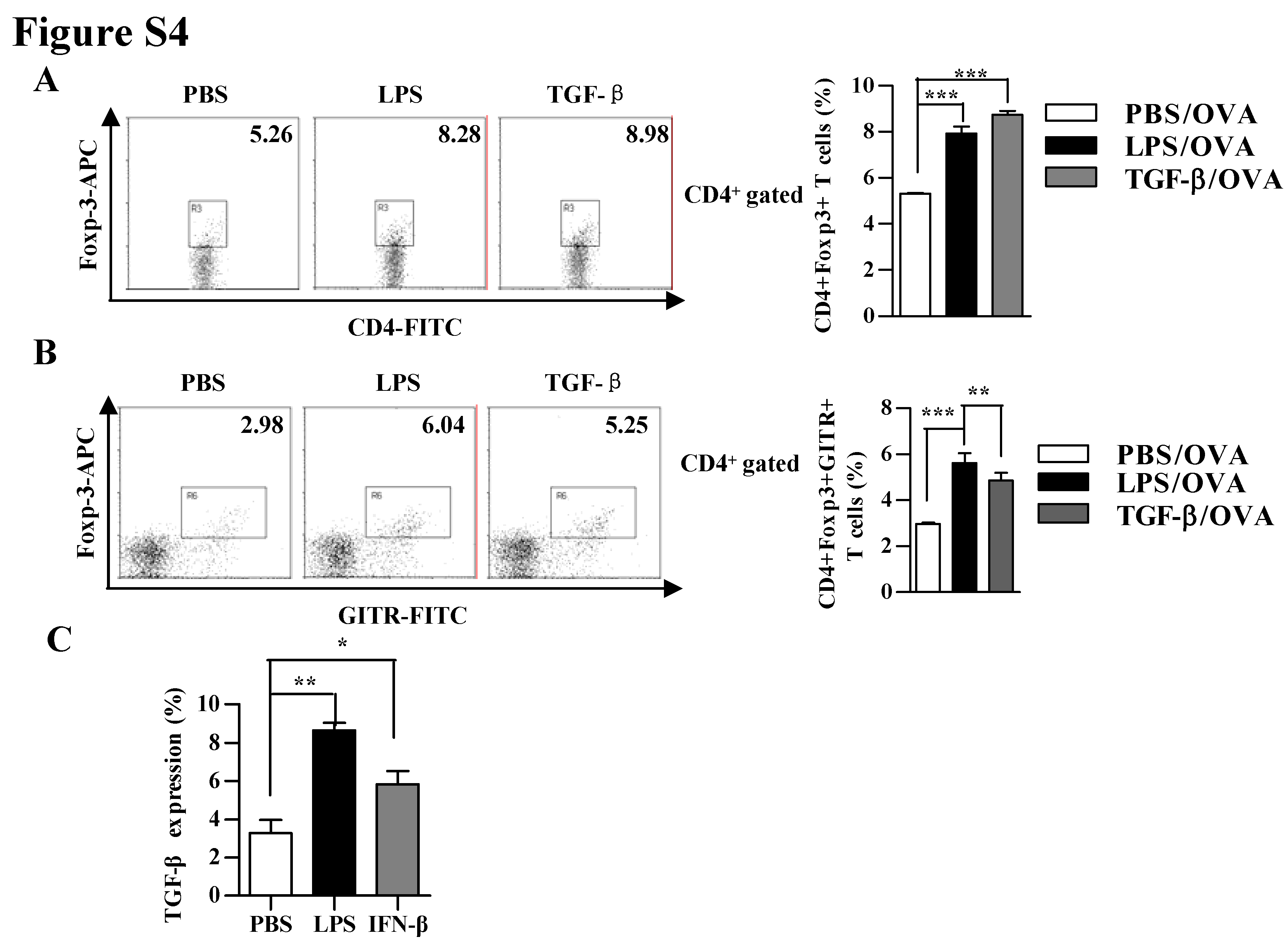

Supplement: Figure S4 — Both LPS and TGF-β increase Treg priming in vitro . (A and B) DCs were treated with LPS (10 ng/ml) or TGF-beta (5 ng/ml) for 12 h which were further conferred OVA pulse at the final concentration of 100 ng/ml. Then, the DCs were coincubated with splenocytes at the ratio of 1∶10. After 3 d co-culture, CD4+Foxp3+ and CD4+GITR+Foxp3+T cell proportion was analyzed by flow cytomentry. Numbers in histogram indicated the positive cells percentages of each analyzed population. Data were given as mean ± SEM, n = 3. **p<0.01, ***p<0.001, one-way ANOVA with post Newman-Keuls test. A representative out of 3 independent experiments was shown. (C) IFN-beta up-regulates TGF-beta expression in DCs. DCs were treated with LPS (10 ng/ml) or IFN-beta (10 ng/ml) for 12 h and the expression of TGF-beta was determined by flow cytometry. Data were given as mean ± SEM, n = 3. A representative out of 3 independent experiments was shown. (TIF) [file pone.0105636.s004.tif]
